# Maximizing the Quality of Non‐Invasive Samples for Conservation Genetics Using Targeted Next‐Generation Sequencing: A Comparison of Fecal DNA Preservation Methodologies

**DOI:** 10.1002/ece3.73772

**Published:** 2026-05-28

**Authors:** Alexis L. Levengood, Katrin Hohwieler, Daniel Powell, Romane H. Cristescu

**Affiliations:** ^1^ Detection Dogs for Conservation, School of Science, Technology, and Engineering University of the Sunshine Coast Sippy Downs Queensland Australia; ^2^ Marine and Terrestrial Megafauna Research Cluster University of the Sunshine Coast Sippy Downs Queensland Australia; ^3^ Centre for Bioinnovation University of the Sunshine Coast Sippy Downs Queensland Australia

**Keywords:** collection methodology, koala, *Phascolarctos cinereus*, sample storage, scat preservation, SNP genotyping

## Abstract

Non‐invasive DNA sampling from feces can provide powerful tools for wildlife research, management, and conservation. However, obtaining high quality and quantity fecal DNA is notoriously problematic, being affected by many different variables. Arguably, the most influential factor within the control of biologists is how samples are collected and stored prior to laboratory analyzes. Here, we aimed to compare different fecal DNA preservation methodologies for their performance with a targeted genotyping approach and improve the quality and quantity of DNA extracted to better inform sampling of feces in the field. We assessed the proportion of missing single nucleotide polymorphism (SNP) data resulting from seven different fecal DNA preservation methodologies on fresh koala ( *Phascolarctos cinereus* ) scats. DNA was successfully obtained from all preservation methodologies; however, optimal recovery of DNA was obtained via a lysis shaken (i.e., washed) methodology, which provided a yield almost equivalent to that of more invasively sampled, high‐quality, tissue samples. Our findings suggest there is a significant advantage of using a lysis buffer and washing technique coupled with targeted genotyping from scats. As a robust sampling method underpins successful data analysis, this optimized fecal sampling technique can further enhance our ability to address critical questions in population ecology, conservation genetics, and population management and help implement improved conservation strategies and decision making.

## Graphical abstract

Obtaining high quality and quantity fecal DNA is notoriously problematic. We compared seven different fecal DNA preservation methodologies for their performance with a targeted genotyping approach to better inform sampling of feces in the field. Optimal recovery of DNA was obtained via a lysis shaken (i.e., washed) methodology, which provided a yield almost equivalent to that of high‐quality, tissue samples.

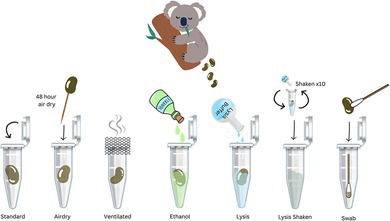

## 1 Introduction

Non‐invasive fecal sampling and genetic analyzes of scats are used increasingly in wildlife research, management, and conservation, proving to be an extremely valuable tool for the study of wild animals (Deiner et al. 2017; Waits and Paetkau 2005; Zemanova 2021). The utility of this methodology allows for estimations of abundance, effective population size, sex ratio, relatedness, home range, habitat use, genetic diversity, gene flow, diet, paternity and mating strategies, and social structure without the need to capture or handle any animals (Byrne et al. 2021; Deiner et al. 2017; Zemanova 2021). Although the non‐invasive nature of scatology has broad appeal, applications are often limited by challenges associated with handling samples for DNA extraction and subsequent analyzes due to poor recovery of host DNA (Morin et al. 2001; Pompanon et al. 2005; Taberlet et al. 1999).

Fecal DNA extraction and amplification success are highly influenced by environmental factors (e.g., temperature, humidity, sun exposure (Brinkman et al. 2010; Murphy et al. 2007; Piggott 2004; Santini et al. 2007)) and diet (Murphy et al. 2007; Panasci et al. 2011), thus factors prior to scat collection, but also by more controllable influences such as age (i.e., time spent in the environment) (Panasci et al. 2011; Piggott 2004; Santini et al. 2007; Schultz et al. 2018) and storage/sample preservation methods (Frantzen et al. 1998; Panasci et al. 2011; Piggott and Taylor 2003; Wasser et al. 1997). Despite many studies attempting to improve fecal DNA genotyping outcomes, with so many variables influencing success, previous techniques developed for other species, or under differing climates/environments, may be of limited applicability to new studies.

Although many variables have been shown to affect the success of DNA acquisition from scats, the single most influential factor in control of most field biologists is how samples are collected and stored prior to transfer to a laboratory (Miles et al. 2015; Piggott and Taylor 2003). As such, several studies have evaluated the relative success of a number of preservation methodologies (e.g., scats stored in ethanol, lysis buffer, DET buffer, silica, dried, frozen, see Tende et al. (2014) for a review) across a range of species (i.e., canids (Miles et al. 2015; Panasci et al. 2011; Piggott and Taylor 2003; Santini et al. 2007)), elephantids (Kouakou et al. 2023), felids (Miles et al. 2015; Tende et al. 2014; Wultsch et al. 2015), marsupials (Miles et al. 2015; Piggott and Taylor 2003), mustelids (Miles et al. 2015), primates (Frantzen et al. 1998; Nsubuga et al. 2004), ungulates (Liu et al. 2014; Soto‐Calderón et al. 2009), and ursids (Murphy et al. 2000, 2002; Wasser et al. 1997; Zhu et al. 2017). Unfortunately, these studies have often reached divergent conclusions, even in cases in which similar preservatives were tested (e.g., Murphy et al. (2002) and Wasser et al. (1997)). This inconsistency underlines that best methods vary among species, as well as possible other variables (e.g., climates). As such many recommend an urgent and on‐going need for studies to rigorously compare commonly used storage types (Santini et al. 2007; Soto‐Calderón et al. 2009; Tende et al. 2014; Wultsch et al. 2015).

Compounding the mixture of conclusions from previous studies, currently all evaluations of fecal DNA preservation methodology have been assessed using microsatellites and polymerase chain reaction (PCR) analyzes. Studies have yet to assess the impact of preservation methodology when using single nucleotide polymorphism (SNP) genotyping. This is surprising, as there has been a surge in conservation and population genetics studies that use SNPs (Galla et al. 2019; Helyar et al. 2011; Hohenlohe et al. 2021; Morin et al. 2004, 2009; Sunde et al. 2020; Willi et al. 2022; Zimmerman et al. 2020), especially those derived from DNA of non‐invasive samples such as scales (Smith et al. 2011), hair (Russello et al. 2015), and scats (Ekblom et al. 2021). Furthermore, the relationship between fecal DNA preservation methodology and targeted SNP genotyping, which promised better recovery of degraded DNA (Hernandez‐Rodriguez et al. 2018; Perry et al. 2010; Snyder‐Mackler et al. 2016), will be of great interest to researchers to identify methodologies leading to further improved genotyping outcomes.

The aim of this study was to identify the optimal fecal DNA preservation methodology from a set of previously described options, using a targeted genotyping approach (DArTCap), which could then inform sampling of feces in the field. Here, we used scats from koalas, a cryptic, arboreal marsupial with a patchy distribution along the east coast of Australia, recently classified as endangered across much of its range (DAWE 2022). Notwithstanding conservation efforts over the last 20 years, koala numbers in Queensland and New South Wales continue to decline, with the extinction of local populations a real possibility (Johnston et al. 2024). Despite a few studies on fecal DNA preservation effectiveness in marsupials, to date no study has compared preservation methods in koalas nor using targeted genotyping methodologies. As such, it is critical that robust preservation methodologies of non‐invasive samples are determined for cutting edge technologies to ensure maximal quality of fecal DNA used in critical conservation and management initiatives (Johnston et al. 2024). We quantitatively compared the effectiveness of seven dry and liquid methodologies for preserving koala fecal DNA. Through an experimental setup we assessed (1) standard scat scrapping (Stenglein et al. 2010), (2) airdried (Piggott and Taylor 2003), (3) ventilated (Carruthers et al. 2019; Wedrowicz et al. 2013), (4) ethanol (Murphy et al. 2002; Tende et al. 2014), (5) lysis buffer (Panasci et al. 2011), (6) lysis buffer shaken (i.e., washing) (Palomares et al. 2002; Panasci et al. 2011), and (7) swabbing (Frantzen et al. 1998). We compared the missing SNP data (i.e., absent SNPs) of these preservation methods to missingness from tissue samples, considered to be a high‐quality sample, to rank various preservation methods' effectiveness for fecal DNA SNP genotyping, using targeted methodologies (DArTCap).

## 2 Materials and Methods

### 2.1 Sample Collection and Fecal Preservation Testing

Veterinary examinations were conducted on 11 wild koalas (six females, five males) from the Gympie region of Southeast Queensland between 2020 and 2021. Biopsy samples for each individual koala were collected in the form of ear tissue, available through standard hole punch for ear tag fitting during examinations (under anesthesia). All biopsies were stored in RNAlater and placed in a −20°C freezer until processing.

**FIGURE 1:**
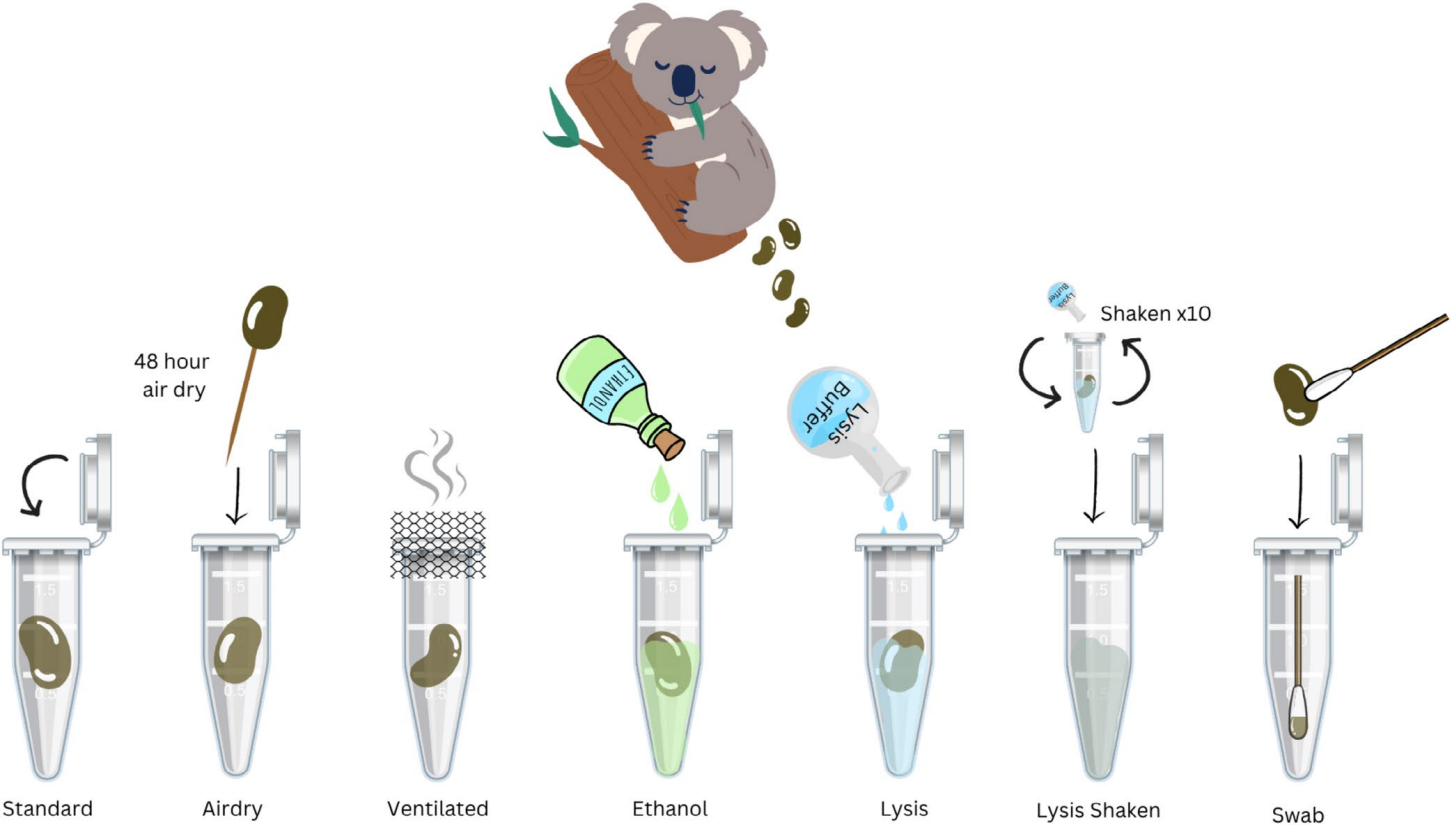
Diagram of fecal DNA preservation methods. Fresh koala scat samples were collected from 11 koalas. Seven pellets for each koala were placed in one of seven treatments (standard, air dry, ventilated, ethanol, lysis, lysis shaken, and swab) within 2 h of defecation. Samples were stored frozen (−20°C) until processing for DNA extraction.

Seven fresh scats were collected from the transport cage of each of these same 11 koalas (Table S1). Scats that appeared wet with urine or crushed were discarded. Scats were sampled using one of seven different fecal DNA preservation methodologies within 2 h of defecation (Figure 1):

1. Standard—a scat was placed in a 5 mL tube with no additional treatment;
2. Airdry—a scat was placed on an individual toothpick embedded into a Styrofoam board and let to air dry in a controlled laboratory for 48 h, then placed in a 5 mL tube;
3. Ventilated—a scat was stored in a 5 mL tube with the cap replaced by a mesh secured around the opening to allow ventilation of volatile compounds;
4. Ethanol—a scat was placed inside a 5 mL tube containing 800 μL of 95% ethanol;
5. Lysis—a scat was placed inside a 5 mL tube containing 800 μL of the QiAamp PowerFecal Pro DNA kit's lysis buffer (CD1 solution, hereafter lysis buffer);
6. Lysis shaken—a scat was placed inside a 5 mL tube containing 800 μL of the QIAamp PowerFecal Pro DNA kit's lysis buffer and shaken by inverting the tube 10 times to loosen and remove the surface layer of the scat. The remaining pellet was then removed using a wooden sterile toothpick and only the lysis wash solution remained;
7. Swab—a dry rayon swab (Copan CLASSIQSswabs) was used to gently scrape all sides of the scat. The scat was discarded, and the swab was stored inside a 5 mL tube.
8. It is worth noting that all liquid preservation methodologies used a volume of 800 μL to reflect the protocol of the DNA extraction kits used. After applying the respective fecal preservation method, sample material was immediately stored in a −20°C freezer until processing (i.e., all sampling collection methods were frozen before DNA extraction), given that non‐invasive samples are generally not processed immediately after collection due to constraints in time and travel involved in accessing field sites. Processing occurred within 1 month of sample collection.

### 2.2 DNA Isolation From Tissue

All DNA extractions were performed by a single researcher to ensure that inter‐individual differences in extraction performance did not unintentionally influence downstream results. DNA was isolated from koala ear biopsies by dicing the tissue into small pieces with a scalpel. DNA was then extracted from these pieces using DNeasy Blood and Tissue kits (Qiagen, Hilden, Germany), following the manufacturer's protocol, with the following variations: after adding proteinase K and vortexing, samples were incubated at 56°C overnight (~10 h) to ensure they were completely lysed. Final DNA isolates were eluted in 200 μL of Buffer AE and concentrated down to a volume of 50 μL using Eppendorf's Vacufuge Vacuum Concentrator (Eppendorf Inc., Hamburg, Germany).

### 2.3 DNA Isolation From Scat Samples

DNA was isolated from exfoliated koala intestinal epithelial cells present on the surface of the scat in the following ways, depending on fecal preservation method. For the standard, airdry, and ventilated samples, DNA was isolated from the surface of the scat by slicing off the outer‐most layer of the scat using a sterile scalpel (Table S1). Additionally, as a potential confounding factor, we weighed the standard scat sample for each individual whole as well as its slices (Table S1). For the ethanol samples, after thawing, the scat was removed, the ethanol mixture was centrifuged at full speed (14,000 rpm), the ethanol was pipetted out, and 800 μL of lysis buffer was added into the tube. For the lysis and lysis shaken samples, the lysis solutions were directly transferred (i.e., the scat was not retained in the samples) to step one of the extraction protocol (see below). Finally for the swab samples, these were transferred directly to step one of the extraction protocol, vortexed for 10 s at full speed, and then the swab was removed from the tube prior to the start of step two.

DNA was extracted from the samples using the QIAmp PowerFecal Pro DNA kit (Qiagen), following the manufacturer's protocol, with the following variations: after adding lysis buffer (step 1), samples were incubated at 65°C for 1 h, and then vortexed for 7 min at maximum speed using a Genie 2 Vortex Mixer (Scientific Industries, New York, US). DNA was eluted in 100 μL of C6 buffer and concentrated to a volume of 50 μL using Eppendorf's Vacufuge Vacuum Concentrator. The purified DNA was stored at −80°C.

### 2.4 SNP Genotyping

One biopsy sample for each individual from the ear tissue and each fecal preservation method (total *N* = 88) was used for SNP genotyping. SNP genotyping was conducted by Diversity Arrays Technology, Canberra, Australia using DArTCap technology. The approach of DArTCap was designed to increase sensitivity and specificity with greater on‐target sequencing coverage (Feutry et al. 2020; Hohwieler et al. 2022). DArTCap involves a combination of complexity reduction methods and next‐generation sequencing platforms (Cruz et al. 2013; Kilian et al. 2012). We utilized koala‐specific capture probes (designed using DNA obtained from koalas in Southeast Queensland) that bind to restriction fragments in the representations carrying the specific DArTseq markers, aiming for approximately 2000 SNP markers. All 11 koalas came from populations from which the capture baits were designed, limiting the number of alleles that may be expected to be absent in more distant populations. Further details regarding SNP genotyping for non‐invasive koala scat through DArTCap can be found in Hohwieler et al. (2022) and Levengood et al. (2025).

### 2.5 Analyzes

The proportion of missing data (i.e., SNP markers not receiving a call during genotyping) was calculated for all samples (*N* = 88), after the removal of monomorphic loci. We ran a binomial generalized linear mixed model (GLMM) using the *lme4* package (Bates et al. 2014) in R version 4.1.1 (R Core Team 2021) to determine whether fecal preservation methods impacted the proportion of missing data in SNP results, compared to high‐quality biopsy samples, when including individual ID as a random effect. We had convergence issues using the proportion of missing SNP data, so instead ran the GLMM with a negative binomial error structure on raw SNP missingness data, without issue. The reference level was set to the biopsy sample (a high‐quality sample for genotyping results) and used to compare all non‐invasive fecal preservation methods.

## 3 Results

**FIGURE 2:**
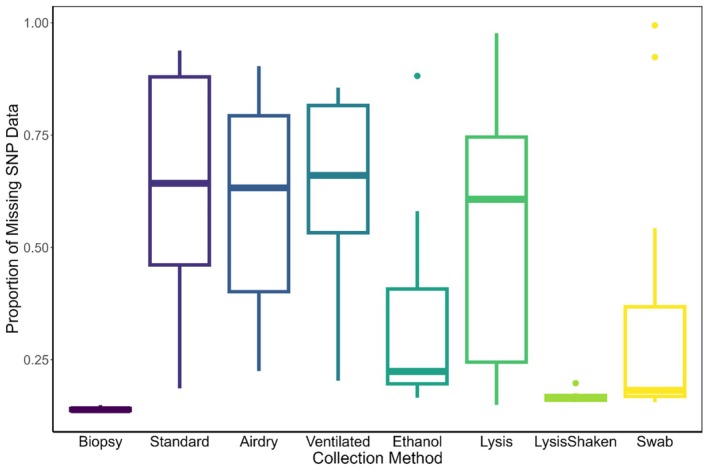
Variation in the proportion of missing SNP data by DNA preservation method (*n* = 88 samples from 11 individuals).

All samples were genotyped and included in the analyzes. Individual samples (including biopsies) varied in their proportion of missing SNP data (range = 13%–99%, $\bar{x}$ = 43%), which we used as a performance metric. The proportion of missing SNP data relates to the number of failed SNP calls from recovered host DNA being either too low in concentration or too degraded to be fully represented. No pattern existed where one individual koala's DNA outperformed another individual (Figure S1). Additionally, no individual koala's scat size (i.e., weight) nor the weight of the scrapings (i.e., surface area) of the scat correlated to the amount of missing SNP data based on methodology (Table S2). However, clear differences emerged between the methodologies tested (Figure 2). As expected, biopsy samples (ear tissue) contained the lowest proportion of missing SNP data among all koalas ($\bar{x}$ = 14%), closely followed by the lysis shaken method ($\bar{x}$ = 17%). Both the biopsy and lysis shaken methods also displayed low variation in the proportion of missing SNP data among individuals (Figures 2 and S1). The ethanol and swab methods (both $\bar{x}$ = 35%) were the next lowest in the proportion of missing SNP data, while lysis ($\bar{x}$ = 55%), airdry ($\bar{x}$ = 59%), ventilated ($\bar{x}$ = 64%), and normal ($\bar{x}$ = 64%) all had relatively high SNP missingness (Figure 2) and large variation among individuals (Figure S1).

**FIGURE 3:**
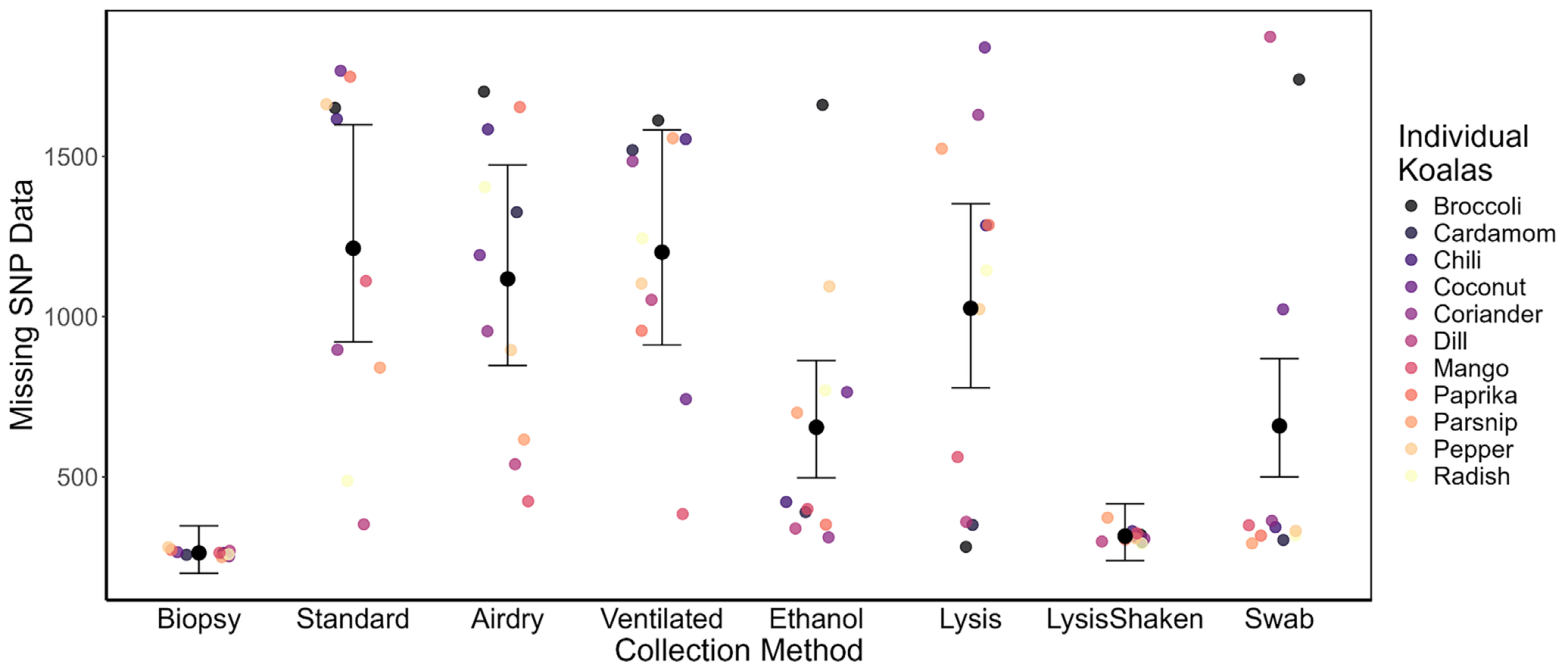
Impacts of DNA preservation method on genetic data quality and variability. Poisson generalized linear mixed model (GLMM) results with the missing SNP data (raw) from extracted koala scats including 95% CIs. Total SNP count =2000. *N* = 88 samples from 11 individuals.

When accounting for individual identity, all non‐invasive scat methodologies performed significantly worse than the biopsy sample (*p* < 0.001), except for lysis shaken which showed no significant difference in the missingness of the raw SNP data (*p* = 0.351, Table 1, Figure 3). In other words, among the non‐invasive scat preservation methods, lysis shaken was superior to the other methods in reducing the amount of missing SNPs and did not perform significantly differently from the high‐quality biopsy (i.e., tissue) samples (range = 85.19%–100% SNP genotype matching, $\bar{x}$ = 98.35% SNP genotype matching, Figures 2 and 3, Table 2).

**TABLE 1:** Results of the effects of DNA preservation method on raw missing SNP data.

Parameter	Estimate	SE	*z*‐value	*p*
Intercept (Biopsy)	5.573	0.142	39.267	< 0.001
Standard	1.517	0.196	7.761	< 0.001
Airdry	1.434	0.196	7.326	< 0.001
Ventilated	1.512	0.195	7.741	< 0.001
Ethanol	0.888	0.197	4.504	< 0.001
Lysis	1.358	0.196	6.945	< 0.001
Lysis Shaken	0.182	0.196	0.932	0.351
Swab	0.894	0.198	4.521	< 0.001

*Note:* Results from a negative binomial generalized linear mixed model (GLMM) with individual ID as the random effect. Intercept represents the biopsy (i.e., high quality) samples. *N* = 88 samples from 11 individuals.

**TABLE 2:** SNP genotype matching comparison between biopsy (i.e., high quality) samples and scats (i.e., non‐invasive) that underwent the lysis shaken methodology, including percent of missing data for both methodologies.

Koala ID	Missing data biopsy (%)	Missing data scat (%)	Similarity (percent genotype match, %)
Broccoli	13.9	16.9	99.7
Cardamom^a^	13.6	15.8	85.2
Chili	13.9	17.6	99.7
Coconut	14.1	16.7	100.0
Coriander	13.4	16.3	100.0
Dill	14.3	15.9	99.9
Mango	14.0	17.2	98.5
Paprika	14.4	16.2	99.9
Parsnip	13.3	19.8	99.0
Pepper	14.9	16.4	100.0
Radish	13.7	15.6	99.9

*Note:* Similarity is calculated from only the matching SNP calls in these data (i.e., missing data was not considered).

^a^ NB: Cardamom was a joey with small scats. Scat size discrepancies can be observed in Table S1.

## 4 Discussion

This study is the first to investigate and evaluate fecal preservation methodologies using a capture probe genotyping approach for non‐invasive samples. Additionally, this study serves as the first reported evaluation of preservation methodologies for koala feces. Importantly, we identified a single, straightforward preservation methodology that optimized fecal SNP genotyping success almost as well as high‐quality tissue samples, which has not been demonstrated prior using other methodologies. These results highlight the potential power of targeted next‐generation genetic approaches (Perry et al. 2010; Snyder‐Mackler et al. 2016), such as DArTCap, when combined with robust preservation methodologies and encourage further optimization for other species. Based on non‐invasive samples from wild koalas, these findings suggest that washing scats using a “lysis shaken” preservation method combined with next‐generation sequencing should allow ecologists to obtain high‐quality genetic data (roughly equivalent to that obtained by tissue samples) yet without capture of animals. Depending on project aims, this could translate into more cost‐effective conservation genetics research, leading to improved mitigation and management outcomes.

Our results showed significant differences in the performance of each fecal DNA preservation methodology, with the exception of one, when compared to the DNA extracted from biopsy tissue. Unsurprisingly, tissue samples yielded the highest quality DNA and remain a gold standard sample type for genetic data quality. The best fecal DNA preservation method, however, the “lysis shaken” method, with the lowest percent of SNP missingness, had only slightly higher SNP missingness than the biopsy method. This result is somewhat surprising as other studies (Panasci et al. 2011; Wasser et al. 1997) that have used lysis buffer to extract fecal DNA were less successful. For example, Panasci et al. (2011) found lysis buffer to be consistently worse than both DET buffer (20% DMSO, 0.25 M EDTA, 100 mM Tris, pH 7.5, and NaCl to saturation) and ethanol and suggested lysis buffers should not be used for future fecal DNA preservation. Additionally, in Wasser et al. (1997) among testing 15 different preservation methodologies, lysis buffers (four tested: Queens lysis buffer, LST buffer, Gerloff lysis buffer, and Qiagen load buffer) performed averagely, and were outcompeted by silica, freeze drying, and desiccation methods. However, in Santini et al. (2007) GUS lysis buffers ranked second behind 95% ethanol stored frozen but were ultimately found to be equally successful as their results were not significantly different. Interestingly all three studies stored the fecal samples in the buffer but lacked details (aside from (Panasci et al. 2011)) in reporting how DNA was then extracted from the samples in the preservation liquids (e.g., whether scats were scrapped, washed, homogenized). It appears, however, that none of these studies using lysis buffer used a washing methodology, which may have been a contributing factor to our successful results. Indeed, Piggott and Taylor (2003) found the washing technique (using SLP buffer or phosphate buffered saline (PBS)) outperformed scraping techniques in macropods and suggested this would be the superior methodology for herbivorous marsupials; however they did not test this with a lysis buffer wash. Additionally, Flagstad et al. (1999) suggested that a surface wash was a crucial step for obtaining higher amplification and lower genotyping error by creating a clean supernatant containing a large number of intestinal cells. It is possible our study combines the best aspects of these two methods and that washing alone (regardless of solution) may be beneficial.

The second most efficient preservation method was comparable between ethanol (involving storage in the medium, discarding the scat, and centrifuging the ethanol mixture out) and swabbing (involving swabbing the outer layer of the fresh scat, followed by vortexing the swab to loosen debris/DNA). Both of these methodologies yielded relatively low SNP missingness, however the swabbing technique generally had lower missingness and less variation between samples with a few (*n* = 2) samples performing extremely poorly. Conversely, the samples stored in ethanol displayed greater variation between individuals and suggest a slightly worse outcome than the swabbing technique. Interestingly, very few studies have compared the effect of swabbing to other methodologies to preserve fecal DNA (though some studies have used a swabbing method without testing (e.g., Ball et al. 2007; Lampa et al. 2008; Rutledge et al. 2009)) and only one study has directly compared swabbing and ethanol storage. Miles et al. (2015) compared swabbing to ethanol storage and contrary to our study found ethanol to be superior in yielding higher DNA concentrations, but also higher inhibition as well. This study, however, was conducted in an arid climate which may have impacted the ability to remove surface fecal DNA using a swab before the mucous layer desiccated. In our study, using very fresh (< 2 h old) koala scats in a humid environment, swabbing seems to be quite effective.

In regard to ethanol, most studies have assessed the effects of ethanol as a preservation medium, with varying successes (high success – (Liu et al. 2014; Murphy et al. 2002; Santini et al. 2007; Tende et al. 2014; Zhu et al. 2017), moderate success – (Kouakou et al. 2023; Panasci et al. 2011; Piggott and Taylor 2003; Wasser et al. 1997), low success – (Frantzen et al. 1998; Santini et al. 2007; Soto‐Calderón et al. 2009; Wultsch et al. 2015)). As such the effectiveness of ethanol as a preservation method seems to vary based on external factors such as species and climate (see Tende et al. (2014) for a review).

The fecal DNA preservation methods that were the least successful were storage in lysis (not shaken/washed), airdried, ventilated, and the standard scraping method, respectively, with high SNP missingness and large variation among individuals. These findings align with many previous studies (Liu et al. 2014; Murphy et al. 2002; Panasci et al. 2011; Piggott and Taylor 2003) where these methods were outperformed by more effective methodologies. Possibly most surprising among this group of poor methodologies is the standard scraping method, which had the greatest proportion of missing SNP data, yet has been used and recommended by many prior (Fernando et al. 2000; Kohn et al. 1999; Livia et al. 2007) and commonly used on koalas (Johnston et al. 2024; Schultz et al. 2018). Additionally, it is interesting that of the methods that performed poorly here, most included “dry methods” as opposed to a liquid‐based preservation methodology. It is not clear why this might be, as previous studies have shown methods such as drying and desiccating to be quite successful (Frantzen et al. 1998; Kouakou et al. 2023; Murphy et al. 2000; Piggott and Taylor 2003; Soto‐Calderón et al. 2009; Wasser et al. 1997), but warrants further investigation.

Targeted genotyping methodologies, such as DArTCap, may allow for greater data recovery depending on the fecal DNA preservation used. Since all previous studies were tested with traditional PCR analyzes (e.g., Wasser et al. 1997; Piggott and Taylor 2003; Soto‐Calderón et al. 2009; Wultsch et al. 2015; Kouakou et al. 2023), it would be ideal to repeat these studies using targeted genotyping to see if the same preservation methodologies yield the best results. It is possible that targeted genotyping approaches provide a more robust genotyping methodology for fecal DNA, depending on the preservation method used, even to a standard similar to high‐quality tissue samples, though additional testing should occur for other species to confirm our results. The likelihood of this is supported by advancements in technology (e.g., the use of a probes design in DArTCap that improves the capture and sequencing of low‐concentration DNA), making it better suited for degraded DNA then shotgun approaches, for example (Hohwieler et al. 2022; Levengood et al. 2025). Further studies utilizing targeted next‐generation methodologies will be critical in improving field and laboratory‐based methodologies moving forward.

Finally, it is also worth noting that there are some limitations to the work conducted here. For example, these results are specific to genetic analyzes of the host, and it is unclear how successful a lysis shaken (i.e., washing) fecal DNA preservation method would be for other DNA molecular work such as establishing diet or detecting pathogens. Future research is needed to assess varying methodologies/techniques depending on the research aim. In saying this, if the research focus is host genes, a lysis wash should be the preferred methodology based on our findings, but further considerations should be taken if additional analyzes are required. For example, the potential of an ethanol shaken methodology could be explored which might combine optimal methods found here for an improved or alternatively successful approach. Additionally, this study used fresh (< 2 h old scats) which did not make contact with soil/litter, nor were the scats exposed to any insects, decomposers, or environmental factors which can influence DNA degradation and potentially impact DNA recovery (Brinkman et al. 2010; Cristescu et al. 2012; Murphy et al. 2007; Piggott 2004; Santini et al. 2007). Although β‐actin has been found to be reasonably stable over time in koala scats (therefore minimizing the impact of age (Levengood et al. 2025)), results may differ depending on the conditions in which the scats are found. Finally, laboratory processing of fresh scats in the field could be impractical for some researchers, and therefore how successful these preservation methodologies would be should the scats be stored frozen before receiving treatment is unknown. Many of these methodologies, and in particular the most successful, lysis shaken, are relatively easy and it would be quite feasible to establish this protocol in the field; however, it would be worth additional testing to see if the success of lysis shaken holds true if the scat is frozen before receiving the treatment of the preservation method.

## 5 Conclusion

Understanding the optimal fecal DNA preservation methodology using targeted genotyping for specific species is critical for improved conservation outcomes and informed decision making. Our findings suggest that a lysis shaken (i.e., washed) fecal preservation method coupled with targeted genotyping can yield koala DNA almost as well as high‐quality sampled tissue. Though our results are specific to koalas, they likely extend to other herbivorous marsupials. Our guidelines should aid in the development of fecal DNA collection in the field and preservation protocols for research and management efforts employing non‐invasive fecal DNA techniques. Non‐invasively sampled scats can provide a powerful tool to obtain estimates of population dynamics (e.g., sex ratio, birth and death rates, immigration, and emigration rates), genetic diversity, and habitat utilization. Furthermore, these data can be used to better inform planning and environmental impact assessment processes, as well as to define evolutionarily significant units and improve genetic management (including animal translocation) of current and future populations.

## Author Contributions

**Alexis L. Levengood:** data curation (lead), formal analysis (lead), investigation (lead), methodology (lead), writing – original draft (lead). **Katrin Hohwieler:** formal analysis (supporting), writing – review and editing (equal). **Daniel Powell:** methodology (supporting), writing – review and editing (equal). **Romane H. Cristescu:** conceptualization (lead), funding acquisition (lead), project administration (lead), writing – review and editing (equal).

## Funding

This work was supported by the Department of Transport and Main Roads, Queensland Government and Redlands City Council.

## Disclosure

Benefit sharing: Benefits from this research accrue from the sharing of our data and results on public databases as described above.

## Ethics Statement

Ethics was granted by the University of the Sunshine Coast (ANA26245).

## Conflicts of Interest

The authors declare no conflicts of interest.

## Supporting information


**Table S1:** Life history information and scat weight for each of the 11 koalas sampled.
**Table S2:** Pearson's correlation (*r*) between proportion of missing SNP data per DNA preservation methodology and scat weight as well as weight of scat scrapings from the scat used for the normal sampling methodology. Biopsy methodology not included. *n* = 11 koalas sampled across seven methodologies.
**Figure S1:** Individual variation between koalas in the proportion of missing SNP data based on DNA preservation method (*n* = 88).

## Data Availability

Data are available within dryad (https://doi.org/10.5061/dryad.hhmgqnkxn). All the required data are uploaded as Supporting Information for review purposes.
