# Supplementary material for: Maximizing the Quality of Non‐Invasive Samples for Conservation Genetics Using Targeted Next‐Generation Sequencing: A Comparison of Fecal DNA Preservation Methodologies
Source: Ecol Evol. 2026 May 28;16(6):e73772. doi: 10.1002/ece3.73772 (PMC13240558; doi:10.1002/ece3.73772)
Supplement: Supplementary file 1 — Table S1: Life history information and scat weight for each of the 11 koalas sampled. Table S2: Pearson's correlation (r) between proportion of missing SNP data per DNA preservation methodology and scat weight as well as weight of scat scrapings from the scat used for the normal sampling methodology. Biopsy methodology not included. n = 11 koalas sampled across seven methodologies. Figure S1: Individual variation between koalas in the proportion of missing SNP data based on DNA preservation method (n = 88). [file ECE3-16-e73772-s001.docx]

**Supplementary Material**

**Title:** Maximizing the quality of non-invasive samples for conservation genetics using targeted next-generation sequencing: a comparison of fecal DNA preservation methodologies

**Authors:** Alexis L. Levengood^1,2^, Katrin Hohwieler^1,2^, Daniel Powell^1,3^, Romane H. Cristescu^1,2, 3*^

**Affiliations:**

1. Detection Dogs for Conservation, School of Science, Technology, and Engineering, University of the Sunshine Coast, Sippy Downs QLD, 4556, Australia
2. Marine and Terrestrial Megafauna Research Cluster, University of the Sunshine Coast, Sippy Downs, QLD 4556, Australia
3. Centre for Bioinnovation, University of the Sunshine Coast, Sippy Downs QLD, 4556, Australia

***Correspondence to:** [rcristes@usc.edu.au](mailto:rcristes@usc.edu.au)

**Table S1**: Life history information and scat weight for each of the 11 koalas sampled.

| Koala ID | Sex | Age Class | Single scat weight (g) | Scat slices weight (g) |
| --- | --- | --- | --- | --- |
| Broccoli | M | Adult | 0.739 | 0.268 |
| Chili | F | Adult | 0.505 | 0.256 |
| Cardamom | F | Joey | 0.315 | 0.090 |
| Coriander | M | Adult | 0.695 | 0.322 |
| Coconut | F | Adult | 0.575 | 0.271 |
| Dill | M | Adult | 0.385 | 0.173 |
| Mango | F | Adult | 0.529 | 0.176 |
| Paprika | F | Adult | 0.319 | 0.214 |
| Parsnip | M | Adult | 0.424 | 0.152 |
| Pepper | F | Adult | 0.264 | 0.120 |
| Radish | M | Adult | 1.368 | 0.339 |


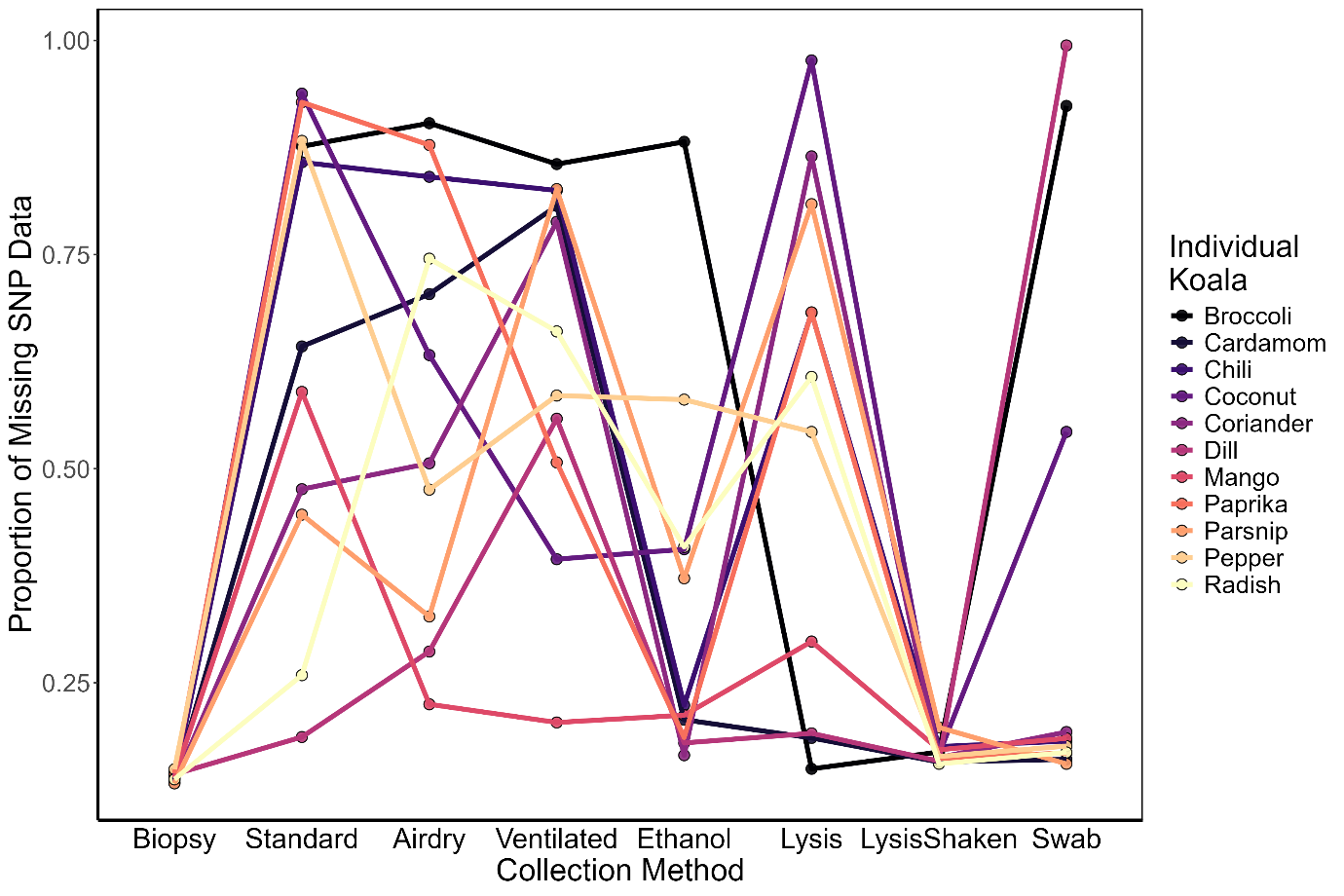


**Figure S1**: Individual variation between koalas in the proportion of missing SNP data based on DNA preservation method (n = 88).

**Table S2**: Pearson’s correlation (r) between proportion of missing SNP data per DNA preservation methodology and scat weight as well as weight of scat scrapings from the scat used for the normal sampling methodology. Biopsy methodology not included. n = 11 koalas sampled across seven methodologies.

| Scat Sample Methodology | Single scat weight (r) | Scat slices weight (r) |
| --- | --- | --- |
| Standard | -0.371 | -0.132 |
| Airdry | 0.242 | 0.317 |
| Ventilated | 0.031 | -0.037 |
| Ethanol | 0.275 | 0.169 |
| Lysis | 0.033 | 0.112 |
| Lysis Shaken | -0.309 | -0.472 |
| Swab | 0.046 | 0.158 |
